# Supplementary material for: Chemosensory continuity from prenatal to postnatal life in humans: A systematic review and meta-analysis
Source: PLoS One. 2023 Mar 30;18(3):e0283314. doi: 10.1371/journal.pone.0283314 (PMC10062646; doi:10.1371/journal.pone.0283314)
Supplement: S1 Table — (PDF) [file pone.0283314.s002.pdf]

S1 Table. Quality assessment of included studies

Non-randomized studies using the ROBINS-I tool

Study	Outcome assessed for risk of bias	Bias due to confounding	Bias in selection of participants into the study	Bias in classification of interventions	Bias due to deviations from intended interventions	Bias due to missing data	Bias in measurement of outcome	Bias in selection of the reported result	Overall bias
Marlier 1998a	Head orientation (duration)	Low	Low	Low	Low	Low	Low	Low	Low
Marlier 1998b	Head orientation (duration)	Low	Low	Low	Low	Low	Low	Low	Low
Hepper 1995	Head orientation (duration)	Some concern	Low	Low	Low	Low	Low	Low	Some concern
Schaal 1995	Head orientation (duration)	Low	Low	Low	Low	Low	Low	Low	Low
Schaal 1998	Head orientation (duration)	Low	Low	Low	Low	Low	Low	Low	Low
Schaal 2000	Negative facial expressions (frequency)	Low	Low	Low	Low	Low	Low	Low	Low
Schaal 2000	Head orientation (duration)	Low	Low	Low	Low	Some concern	Low	Low	Some concern
Schaal 2000	Mouthing behaviour (duration)	Low	Low	Low	Low	Low	Low	Low	
Wagner 2019	Mouthing behaviour (duration)	Low	Low	Low	Low	Low	Low	Low	Low

S1 Table. *(continued)*

Randomized studies using the ROB 2 tool							
**Study**	**Outcome assessed for risk of bias**	**Bias arising from the randomization process**	**Bias due to deviations from intended interventions**	**Bias due to missing data**	**Bias in measurement of outcome**	**Bias in selection of the reported result**	**Overall bias**
Mennella 2001	Negative facial expressions (frequency)	Low	Low	Low	Low	Low	Low
