# Supplementary figures and images for: Chemosensory continuity from prenatal to postnatal life in humans: A systematic review and meta-analysis
Source: PLoS One. 2023 Mar 30;18(3):e0283314. doi: 10.1371/journal.pone.0283314 (PMC10062646; doi:10.1371/journal.pone.0283314)

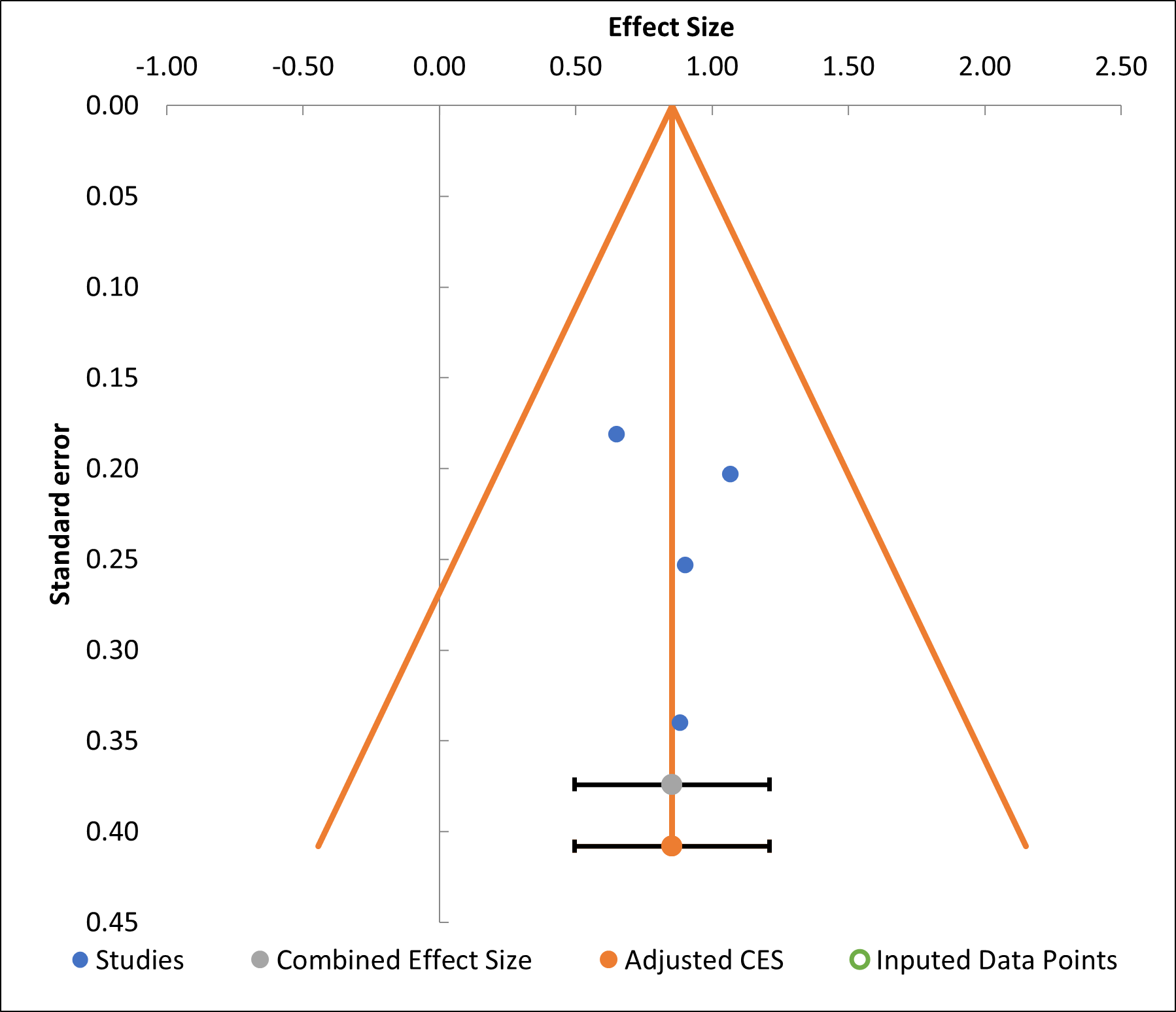

Supplement: S1 Fig — (TIF) [file pone.0283314.s003.tif]
